# Citrulline malate supplementation does not improve German Volume Training performance or reduce muscle soreness in moderately trained males and females

**DOI:** 10.1186/s12970-018-0245-8

**Published:** 2018-08-10

**Authors:** Andrew J. Chappell, Daniel M. Allwood, Rebecca Johns, Samantha Brown, Kiran Sultana, Annie Anand, Trevor Simper

**Affiliations:** 1 Food and Nutrition group, Sheffield Business School, Sheffield Hallam University, Stoddard Building, City Campus, Sheffield, UK; 2 Department of Biosciences and Chemistry, Sheffield Hallam University, Owen Building, City Campus, Sheffield, UK

**Keywords:** Anaerobic exercise, Isometric force, Lactate, Return of force, Muscle soreness, GVT, Citrulline malate

## Abstract

**Background:**

Use of supplements to aid performance is common practice amongst recreationally active individuals, including those without a sufficient evidence base. This investigation sought to assess whether acute supplementation with 8 g of citrulline malate (CM) (1.11: 1 ratio) would improve anaerobic performance.

**Methods:**

A randomised double blind placebo control trial was employed, using a counterbalanced design. We recruited recreationally active men and women to take part in an isokinetic chair protocol, based on German Volume Training (GVT) whereby participants attempted to perform 10 sets of 10 repetitions against a force representing 70% of their peak concentric force.

**Results:**

The number of repetitions achieved over the course of the GVT was 94.0 ± 7.9 and 90.9 ± 13.9 for placebo and CM respectively. There was no significant difference between the placebo and CM treatment for number of repetitions (*P* = 0.33), isometric (*P* = 0.60), concentric (*P* = 0.38), or eccentric (*P* = 0.65) peak force following the GVT. Total muscle soreness was significantly higher in the CM compared to the placebo treatment following the GVT protocol over 72 h (*P* = 0.01); although this was not accompanied by a greater workload/number of repetitions in the CM group.

**Conclusions:**

We conclude that an acute dose of CM does not significantly affect anaerobic performance using an isokinetic chair in recreational active participants. Practical implications include precaution in recommending CM supplementation. Coaches and athletes should be aware of the disparity between the chemical analyses of the products reviewed in the present investigation versus the manufacturers’ claims.

**Electronic supplementary material:**

The online version of this article (10.1186/s12970-018-0245-8) contains supplementary material, which is available to authorized users.

## Background

Citrulline malate (CM) is an organic salt made up of the non-essential amino acid _L-_citrulline and _L-_malic acid, an intermediate in the citric acid cycle. The main dietary source of citrulline is watermelon (*Citrullus vulgaris*), while malic acid is common to apples (*Malus pumila*) and grapes (*Vitis vinifera*). Initially CM was utilised as a pharmaceutical drug (STIMOL©, BIOCODEX Gentilly, France), for the treatment of patients suffering from asthenia to mitigate recovery time following physical activity [1]. However, the consistent search by athletes to obtain a competitive advantage has led to the use of CM as a potential ergogenic aid. Citrulline malate has been purported to improve performance in both aerobic and anaerobic exercise modalities via a variety of mechanisms including: improved ammonia and lactic acid metabolism, increased oxygen delivery capacity via increased vasodilation and increased adenosine triphosphate production via increases in kreb cycle intermediates [2, 3].

Citrulline malate has been shown to increase lactate and ammonia clearance following exercise [4, 5]; this is via acting as a nitrogen iron acceptor during the first stage of the urea cycle [6]. Supplementation may therefore improve ammonia-buffering capabilities reducing acidosis which occurs during intense exercise, as well as reducing muscle soreness post exercise [7]. Secondly, citrulline can be synthesised to arginine (ARG), a precursor to arginine succinate (AS), and nitric oxygen (NO). The ensuing conversion of AS to ARG by argininosuccinatelyase results in the formation of fumarate an intermediate in the citric acid cycle [8]. The conversion of citrulline to ARG via AS is thought to be the rate limiting step in the NO cycle [9], and supplementing with CM has been demonstrated to increase blood ARG and ornithine [10, 11]. By way of comparison, citrulline is more effective in increasing plasma ARG, as a large proportion of ARG is lost to splanchnic extraction, used as a substrate for ureagenesis or degraded to yield ornithine and proline [12]. Arginine itself is a known vasodilator and CM indirectly increases vasodilation and production of nitrogen oxygen synthase (NOS) in response to supplementation, which may have a performance enhancing effect [11, 13].

Supplementation with CM has also been shown to affect the rate of oxidative ATP production in response to exercise [2]. This effect has been attributed to the malic acid portion of the supplement as an intermediate utilised in the citric acid cycle. Only a single study has measured performance using citrulline without malate which indicated a reduction in time to exhaustion following an incremental treadmill test to exhaustion [14]. It is not inconceivable that malate may have a potential role in increasing aerobic exercise performance in combination with citrulline, however it should be noted that this hypothesis is predicated on a limited number of studies. Citrulline malate’s potential ability to improve skeletal muscle metabolism and/ or improve efficiency would be expected to improve overall exercise performance and resistance to fatigue. A comprehensive review of citrulline and NO metabolism is beyond the scope of this paper, however interested readers are directed to reviews by Curis et al. [3] and Besco et al. [15].

The ability to resist fatigue and consequently improve performance/recovery would be of value to athletes. Studies investigating the effect of CM on exercise performance in humans and animals are not without precedent and there are numerous papers suggesting the positive effects of acute CM dosages between 3 and 12 g on both aerobic and anaerobic performance related to muscle damage, fatigue resistance and muscle soreness [2, 14, 16–23]. However, a cause and effect relationship between CM supplementation and exercise performance/recovery has yet to be established, positive findings require corroboration, while a number of recent investigations have failed to demonstrate efficacy of CM [24–27]. Furthermore, a position stance from the International Society of Sports Nutrition on CM states it is unclear whether adding these types of nutrients to energy drinks or sports supplements provides an additive effect over carbohydrate or caffeine commonly found within sport supplement formulas [28]. There is therefore a need for further testing of CM to justify inclusion in both resistance and endurance training supplementation protocols. The effect of CM has on muscular endurance and loss of force utilising an isokinetic protocol is yet to be tested. The aim of this investigation was to assess subjects’ ability to resist fatigue during resistance exercise following an acute dose of CM using a randomised double-blind placebo crossover trial with, the main outcome measures of: total number of repetitions, muscle soreness, and blood lactate. A German Volume Training (GVT) protocol was selected as a means to elicit anaerobic fatigue because of its repeated bout nature, and the difficulty of completing the protocol at selected intensity. German Volume Training also provides a clear outcome measure associated with measuring fatigue (e.g. number of complete repetitions) as well as a real world similarity with programmes used by resistance trainers likely to benefit from CM. We hypothesise that CM will have an effect on exercise performance.

## Methods

### Study design

**Fig. 1:**
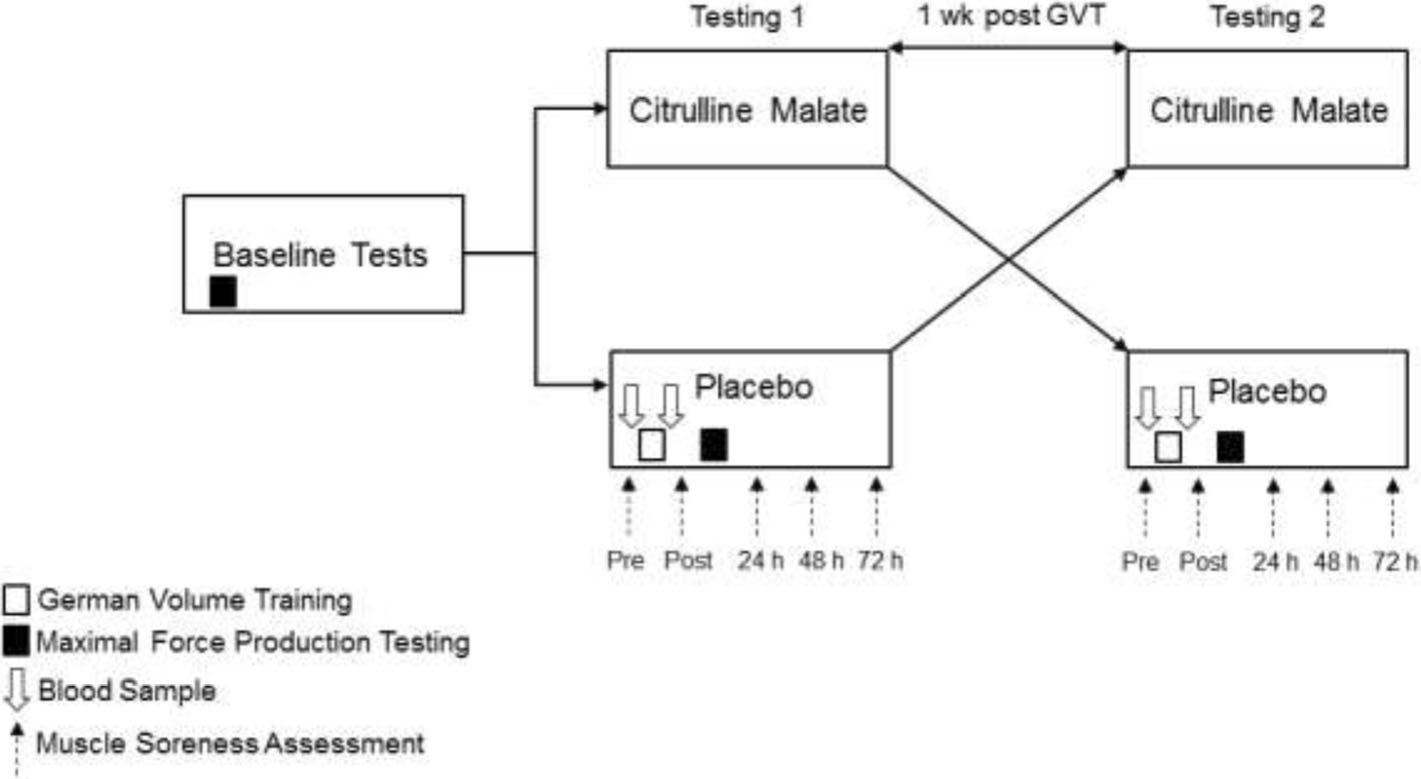
Experimental Design. Each subject visited the laboratory one week apart. Subjects were supplemented with either 8 g of citrulline malate or a placebo. The German Volume Training (GVT) was performed utilising an isokinetic single leg, leg extension protocol, 10 sets of 10 repetitions at 80% 1 RM (obtained during baseline testing), with 1 min rest between sets

A randomised double-blind placebo cross-over design was implemented (Fig. 1). Each subject reported to the laboratory on three separate occasions 7 days apart. Subjects were randomised with a counterbalanced approach to either treatment or placebo using a random number generator and concealment was performed by an independent researcher (MB). Blinding was revealed on completion of the trial. On the first visit subjects were familiarised with the experimental conditions, and the visual analogue muscle soreness scale (VAS). During the first visit, subjects performed the baseline maximum strength determinations for isometric and dynamic concentric/eccentric contractions using an electromechanical dynamometer (System 4 Pro, Biodex Medical Systems, New York, USA). The dynamometer was set up to measure peak force (F_max_) generation for the knee extensor muscles. The same warm up protocol was used for each visit to the laboratory. Following a warm up, subjects performed 3 isometric, concentric and eccentric maximum voluntary contractions (MVC) per set, for 3 sets with 3 min rest between sets. The highest peak torque was recorded for each contraction. The peak torque achieved during the dynamic concentric contraction was used to calculate the resistance used for the GVT protocol, set at 70% concentric F_max_. On the second and third visits, subjects provided a blood sample and were supplemented with either 8 g of CM (1.11: 1 ratio) or placebo, completed a 24 h dietary recall and muscle soreness scale. After an hour subjects provided an additional blood sample followed by performing the GVT protocol of 10 sets of 10 repetitions, with a one minute rest between sets. The total number of repetitions was counted across each set and failure was determined when a full range of motion could no longer be completed. Following the GVT an additional blood sample was taken from the subject after which eccentric, concentric and isometric strength were retested. After the trial subjects completed the muscle soreness scale as well as at 24, 48 and 72 h. The following week subjects reported to the lab to complete the trial under the opposite treatment condition.

### Subject characteristics

Eighteen subjects (13 male, 5 female) volunteered to participate in this study. Two males dropped out citing time commitments, while one female dropped out for reasons unrelated to the study. The subject characteristics are displayed in Table 1. Subjects were recruited from Sheffield Hallam University campus and gyms in the surrounding area via posters, electronic advertising across the university network and word of mouth. All subjects were healthy, not suffering from any underlying health conditions, free from injury, non-smokers and not currently using medication. Subjects were advised to follow their normal dietary and supplementary regime for the duration of their involvement in the trial (as both stopping existing and beginning new supplements may have interfered with results). Subjects were advised to avoid any strenuous exercise 48 h pre and post visits to the laboratory. All subjects were following a structured resistance training programme at least twice per week, for at least 6 months. After explanation of all the experimental procedures written consent was obtained from all participants prior to taking part in the trial; which was approved by the Sheffield Hallam University Food Research Ethics committee.

**Table 1:** Baseline subject characteristics

	*n* - 15
Sex	11 M, 4 F
Age (yrs)	23.67 ± 2.41
Weight (kg)	75.15 ± 13.67
Height (m)	1.72 ± 0.10
Fat Mass (%)	14.91 ± 5.99
BMI (kg/m^2^)	25.16 ± 3.19

±, indicates standard deviation; *BMI* Body mass index, *yrs.* years, *M* male and *F* female

### Anthropometrics and muscle soreness scale

During the initial visit to the laboratory anthropometric measurements were taken. Height and weight were assessed using a stadiometer (Holtain, Crymych, United Kingdom), and electronic scales (InBody 720, Biospace, Urbandale, Iowa, USA). Body composition was established via bioelectrical impedance (InBody 720) and body mass index calculated based on kg/m^2^. Subjects were familiarised with the VAS. A demonstration of how to palpate and score muscle soreness was provided. Muscle soreness was scored between 0 to 100 (0 indicated no pain, and 100 worst pain imaginable) corresponding to four sites on the quadriceps: the Vastus Medialis, Vasus Lateralis, Retus Femoris and Tensor Fasciae Latae. Muscle soreness was measured pre and post GVT and subjects were provided with an internet link to complete the VAS (Qualtics, Provo, Utah, USA) at 24, 48 and 72 h after exercise. Subjects were prompted by email and text message prompts to complete the questionnaire.

### Warm-up and cool-down

Prior to taking part in exercise, all subjects performed the same warm-up. The warm-up, consisted of 10 min of cycling at a rating of perceived exertion of 12 (Borg scale 6–20), followed by 3 sets of 10 repetitions, of concentric only unilateral knee extensor exercises performed on the dominant leg. Repetitions were performed over a 90° range of motion; from the furthest point a subject could fully flex their working leg with a resistance of 20, 30, and 40 N m. An additional 5 min was then allocated to subject to engage in unsupervised stretching. The cool down consisted of 10 min of cycling at 8 on the perceived exertion scale, and followed the muscle soreness assessment.

### Assessment of maximal force production

After a warm-up, unilateral quadriceps maximal voluntary torque was measured for isometric, concentric and eccentric conditions. All testing was performed on the subject’s dominant leg using an electronic dynamometer (Biodex, System 4 Pro, Shrley, New York, USA). Isometric contractions were maintained for 5 s at 90° knee flexion angle with 20 s rest between contractions. The dynamometer movement arm was positioned with the centre of rotation adjacent to the pivot point of the knee (corresponding to the femoral condyles of the femur), and adjusted to 90° using a goniometer. For concentric and eccentric conditions a 90° (± 1°) range of motion based on a subject’s ability to maximal flex and contract their quadriceps was utilized. Limb weight was recorded at the extremes of the subject’s range of motion. Sets of 3 concentric and eccentric contractions were performed for 3 repetitions at 60°·^second-1^, with the eccentric contractions performed in a passive manner. Three minutes recovery was allocated between each test and the highest F_max_ recorded using a computer (Dell, Round Rock, Texas, USA).

### German volume training

Subjects were asked to refrain from strenuous exercise 48 h prior to and after each GVT session. All GVT sessions were performed at the same time of day (± 1 h), 7 days apart, and under the same laboratory environmental conditions (21 °C, 45–55% RH). Following the warm-up the GVT was performed consisting of 10 sets of concentric only single leg knee extensor exercises at 70% of the subjects concentric F_max_. Subjects performed a maximum of 10 repetitions per set with 1 min recovery between sets. Repetitions were performed over a 90° range of motion and the set ended when the subject could no longer complete a full range of motion. The number of complete repetitions performed for each set was recorded. Following the GVT maximal force production and muscle soreness was reassessed. Subjects were provided with verbal encouragement throughout the GVT session. Subjects repeated the GVT protocol under both treatment conditions.

### Blood sampling and lactate analysis

Prior to the GVT exercise protocol, subjects rested for 10 min in a seated position. Blood samples were obtained by way of finger prick using a lancet (Accu-chek, Safe-T-Pro). A 20 цl blood sample was taken using a capillary tube and added to an eppendorf containing heperin and saline. Lactate was read immediately using a Biosen C-line (EKF diagnostics, Ebendorfer, Germany). A second blood sample was taken for lactate analysis within 1 min of the completion of the GVT protocol.

### Supplementation

Subjects were provided with either the treatment (8 g of Citrulline Malate 1.11:1 ratio, 4.21 g citrulline, 3.79 g malate (Bulk Powders, United Kingdom), or placebo (6 g of Citric acid (Sigma-Aldrich, Dorset, United Kingdom). Both conditions were provided in a mixture of 70 ml of fruit cordial and 150 ml of water by RJ, or AA, and were consumed within a 5 min under the supervision. Drinks were prepared in advance by JG based on information provided by MB, all authors were blinded for the duration of the trial period. The dosages and timing of the drink provision were based on research, which show citrulline peak levels occur 1 h after administration [10].

### Dietary intake and analysis

Prior to the GVT, subjects were asked to recall all the food and drink they consumed in the past 24 h. Subjects were advised to refrain from starting any new dietary or supplement regimes for the duration of their involvement in the project and to consume similar foods the day before and prior to visiting the laboratory. The contents of the 24 h recall were analysed using dietary analysis software (Nutritics 2017, Dublin, Ireland). All results are expressed as total grams and calories per kilogram of bodyweight.

### Determination of supplement quality

The ratio of citrulline to malate within the dietary supplement used in the trial was assessed using nuclear magnetic resonance (NMR) spectroscopy. By way of comparison, 5 commercially available CM supplements with purported 2:1 ratios were also assessed from the following supplement companies: Trade Ingredients (South Shields, United Kingdom), Peak Supps (Bridgend, United Kingdom), Bodybuilding Warehouse™ (Manchester, United Kingdom), MyProtein™ (Northwich, United Kingdom) Bulk Powders® (Colchester, United Kingdom). The CM utilised by the participants in the present investigation was obtained from Bulk Powders ®. The analysis was performed individually on each of the five supplement powders in a blinded protocol and each powder was analysed in triplicate. A standard NMR tube containing 100 mg of the citrulline malate supplement powder was added to 1 mL of D_2_O (Fisher Scientific, UK) for analysis. The tube was warmed to 40 °C and agitated to ensure complete dissolution of the solid and therefore analysis of the total organic fraction of the sample. Once the solid was completely dissolved, the solution was allowed to cool to room temperature and analysed on a Bruker Avance DPX-400 NMR spectrometer operating at resonance frequencies of 400 MHz (^1^H) and 100 MHz (^13^C). Initially, all ^1^H and ^13^C NMR chemical environments in both citrulline and malic acid were unambiguously assigned using ^1^H, ^1^H–COSY, ^13^C, DEPT–135, HSQC and HMBC experiments. The ^1^H NMR signals were then integrated, with the signal resulting from H9 on malic acid (at ∂ = 4.37 ppm) being calibrated as 1. The integration values for the triplicate runs of all five supplements are available as supplemental additional files (Additional file 1). All possible comparisons of integral values between chemical environments in citrulline and those in malate were then performed. These results are summarised in as additional files (Additional file 2). The mean nuclear ratios for each individual replicate were then compiled into overall mean and standard deviation values which represent the citrulline:malate molar ratio calculated from 8 individual data comparisons and three total experimental repeats.

### Statistical analyses

An a priori power calculation was performed (G* power version 3.1.9.2) based on the main outcome of number of repetitions; in order to detect a difference between the two-dependent means for the total number of repetitions (matched pairs) with a power .80 and alpha 0.05 which determined a sample size of 14 people for the crossover design utilised. We analysed differences in means between lactate and total number of reps performed using a paired samples t-test. Differences between reps performed over sets during the GVT protocol and muscle soreness over days was assessed using a repeated measures analysis of variance (RMANOVA). Mauchly’s test of sphericity was applied to the data to examine if sphericity was violated and if this was the case the Greenhouse-Geisser estimate was utilised. Post Hoc test were performed using pairwise comparisons with Bonferroni adjustment. Difference between F_max_ means between baseline, and both treatment conditions was assessed using a one way ANOVA.

## Results

### Dietary intake

No side effects were reported and the supplement and placebo were well tolerated. The dietary intake from the 24-h recall is presented in Table 2, there was no significant difference for macronutrient or energy intake between treatment conditions. Protein and carbohydrate intakes were within current ACSM/ISSN guidelines for strength and power athletes (Protein, 1.6 to 2.0 g/kg bw, CHO 4 to 7 g/kg bw). Mean fat intake was similar to the ACSM/ ISSN guidance (20 to 30%). Mean alcohol intake over the two treatment conditions was negligible (0.05 g/kg bw).

**Table 2:** Subjects 24 h dietary intake preceding German volume training for both treatment arms

	Citrulline Malate	Placebo	*P* Value	Mean Intake
Protein g/kg bw	2.7 ± 3.0	3.0 ± 2.4	0.39	2.9 ± 2.58
Carbohydrate g/kg bw	5.5 ± 4.9	6.6 ± 4.2	0.30	6.1 ± 4.4
Fat g/kg bw	3.1 ± 4.1	1.1 ± 0.6	0.07	2.1 ± 2.6
Total Energy kcal/kg bw	26.9 ± 6.6	27.6 ± 9.5	0.81	27.2 ± 8.4

±, indicates standard deviation; g/kg bw for macronutrient and kcal/kg bw for total energy intake scaled for bodyweight. Results analysed using student t-test

### German volume training performance

**Fig. 2:**
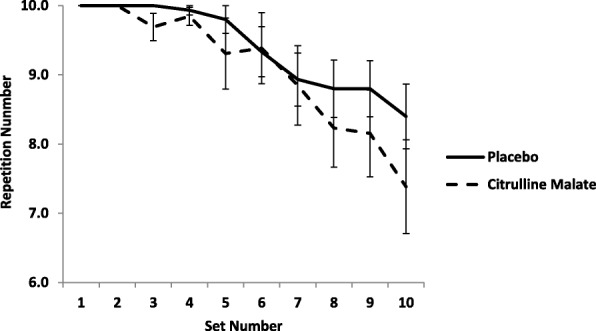
Mean Number of Repetitions Performed Per Set, with and without Citrulline Malate Supplementation. Means were compared using a repeated measures ANOVA, No significant difference was detected between placebo and treatment *P* = 0.33, or treatment × sets *P* = 0.34. Repetitions significantly decreased over the ten sets *P* = 0.01, e.g. subjects performed significantly less repetitions as they progressed from set 1 to set 10 irrespective of treatment. Error bars indicate standard error of the mean

Subjects’ GVT performance data is displayed in Fig. 2. There was a significant difference in the number of repetitions achieved over the ten sets (time *P* = 0.01). The participant’s ability to complete a full set of 10-repetitions progressively declined from set three. The mean number of repetitions performed by the placebo and CM group declined to 8.0 ± 0.6 by set ten. There was no significant difference between the placebo and CM groups for repetition performance (*P* = 0.33) and no interaction between treatment × sets (*P* = 0.34). The mean number of total repetitions performed across the treatment conditions was 92.5 ± 11.5 from a possible 100 repetitions. There was no significant difference (P = 0.34) in the total number of repetitions performed between treatments (94.0 ± 7.9 Placebo, vs 90.9 ± 13.9 CM). Pairwise comparisons indicated there was a significant difference (*P* < 0.05) in the number of repetitions achieved during the GVT between sets 3, 5, 6, 7, 9 and 10. For more detail on pairwise comparisons, see additional files (Additional file 3).

### Maximal force production

Maximal isometric, concentric and eccentric force at baseline and following each treatment condition is presented in Table 3. A total of 10 subjects completed the F_max_ follow-up tests due to technical issues. There was no significant difference between isometric (*P* = 0.60), concentric (*P* = 0.38) and eccentric (*P* = 0.65) F_max_ following the GVT with either the placebo or CM treatment. Moreover, no significant differences were noted in the decline of isometric (CM: − 5.61 ± 47.25 N, Placebo: 18.89 ± 50.64 N, *P* = 0.25), concentric (CM: − 13.59 ± 56.68 N, Placebo: − 10.38 ± 56.57, *P* = 0.76), or eccentric (CM: − 4.02 ± 51.17 N, Placebo: − 2.19 ± 50.65, *P* = 0.64) F_max_ (Table 4).

**Table 3:** Maximal voluntary force production (N) at baseline and following German volume training with either citrulline malate or placebo

	Baseline	Citrulline Malate	Placebo	*P* Value
Isometric	208.2 ± 70.9	213.1 ± 80.3	206.2 ± 55.4	0.37
Concentric	142.4 ± 60.0	144.6 ± 62.1	159.2 ± 53.8	0.96
Eccentric	142.6 ± 77.1	135.8 ± 77.7	146.9 ± 66.9	0.99

±, indicates standard deviation. Means compared using a one-way ANOVA. Results based on n - 10

**Table 4:** Ratios of citrulline to malate from different supplement companies with purported 2:1 ratios measured by nuclear magnetic resonance spectroscopy

Company	Citrulline: Malate Ratio	Citrulline: Malate g / per 8 g dose
TRADE INGREDIENTS	1.92: 1 ± 0.10	5.26: 2.74
PEAK SUPPS	1.62: 1 ± 0.12	4.95: 3.05
BODYBUILDING WAREHOUSE™	1.57: 1 ± 0.15	4.88: 3.12
MYPROTEIN™	1.51: 1 ± 0.04	4.81: 3.19
BULK POWDERS®^a^	1.11: 1 ± 0.02	4.21: 3.79

±, indicates standard deviation; TRADE INGREDIENTS (South Shields, United Kingdom), PEAK SUPPS (Bridgend, United Kingdom), BODYBUILDING WAREHOUSE™ (Manchester, United Kingdom), MYPROTEIN™ (Northwich, United Kingdom) BULK POWDERS® (Colchester, United Kingdom)

^a^The citrulline malate used in the present investigation was obtained from BULK POWDERS®

### Blood lactate and delayed onset muscle soreness

**Fig. 3:**
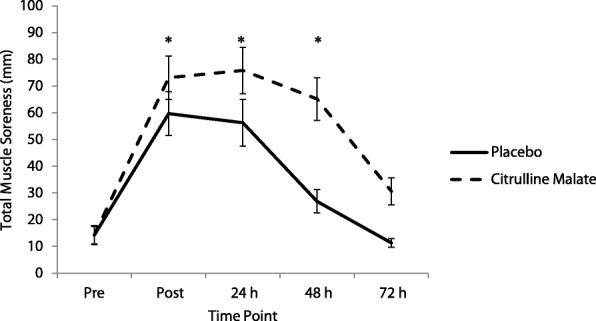
Quadriceps Muscle Soreness Over Time following German Volume Training with, and without Citrulline Malate. Means were compared using a repeated measures ANOVA. There was a significant difference between treatment, time and treatment × time *P* < 0.01. i.e. significantly more muscle soreness was observed in the citrulline malate treatment compared to the placebo treatment over time. A Post Hoc was performed using a pairwise comparison and Bonferroni adjustment. * symbols indicate significant difference between pre and 72 h time point with. Error bars indicate standard error of the mean, h, hours

Blood lactate concentrations measured pre GVT in the Placebo and CM treatment were 1.45 ± 0.54 mmol/l and 1.69 ± 1.07 mmol/l respectively (*P* = 0.53). Following the GVT protocol blood lactate increased to 4.16 ± 1.34 mmol/l and 4.31 ± 1.31 mmol/l in the placebo and CM treatment groups respectively (*P* = 0.72). The difference between pre and post GVT blood lactate concentrations was calculated. There was no difference (*P* = 0.48) between treatment conditions (CM: 2.51 ± 1.53 mmol/l vs Placebo 2.87 ± 1.25 mmol/l). The effect of the GVT protocol on the sum of total muscle soreness is displayed in Fig. 3. Muscle soreness increased significantly over time (*P* < 0.01). Muscle soreness increased immediately following the GVT protocol and peaked at 24 h post exercise before declining by 72 h period in both treatment groups. Pairwise comparisons indicated soreness was significantly higher (*p* < 0.05) post GVT and at 24 and 48 h, compared to the pre and 72 h measurement (see Additional file 4). Total muscle soreness over 72 h was significantly higher in the CM compared to the Placebo treatment following the GVT training protocol (*P* < 0.01). There was also a significant interaction between treatment × soreness (*P* = 0.01). There was a significant difference in soreness ratings between the four muscle sites palpated and assessed (*P* = 0.01). Analysis of specific muscle site soreness indicated that the Vastus Medialis and the Vastus Lateralis sites had consistently higher soreness ratings throughout the trial compared with the Tensor Fasciae and Rectus Femoris. Site specific VAS scores are available as an additional file (Additional file 5).

### Supplement analysis

The ratio of citrulline to malate measured in formulas from different supplement companies with purported 2:1 ratios is displayed in Table 4. The CM supplement used in the present investigation was supplied by Bulk Powders® (Colchester, United Kingdom).

## Discussion

We measured the effects of an acute 8 g dose of CM on anaerobic performance/recovery using a GVT protocol performed with an isokinetic chair in 15 subjects. The acute dose of CM was administered one hour before resistance training and did not significantly affect the number of repetitions performed. This finding is contrary to similar studies, which found significant effects from CM of the same dose [18, 19, 22]. In the present investigation not only did the CM not attenuate the marker of muscle soreness, but was associated with greater soreness over the 72 h following exercise. Moreover, CM supplementation had no effect on blood lactate concentrations following exercise. More recently, a trial using a lower dose (6 g versus 8 g in the present investigation) did not find any significant difference in any dependent variables (including number of repetitions, soreness, creatinine kinase levels and rating of perceived exertion [25]. The present investigation also included chemical analysis of the supplement used, which concluded that the ratio of citrulline to malate was not equivalent to the ratio stated by the manufacturer. Analysis suggests that the supplement contained just over half the manufacturers stated dose of citrulline. Subsequent analysis of four additional commercially available CM products, with a purported ratio of 2:1 (citrulline to malate), shows that only one of these products had a value close to the ratio stated by the manufacturers. This is the first study, to our knowledge, to report the citrulline to malate ratio of a number of these supplements.

Although no difference was found for the main outcome measure of repetitions, this design utilised an acute dose of CM and our data supports the findings of recent investigations of resistance training performance [25–27]. In each instance, a 6 to 8 g acute dose CM had no effect on the number of repetitions achieved when performing either upper or lower-body resistance exercises compared to a placebo. By way of comparison, our results are in contrast to other trials, which investigated the effects of 8 g of acutely administered CM on the number of repetitions achieved during both upper and lower body exercise challenges [17, 18, 22]. It is however worth pointing out that there are differences in the study design, subject populations and mode of delivery between the present investigation and previous trials. The present study and utilised a single-leg leg extension exercise, and although the subjects had some experience with weight training the lack of familiarisation with the isokinetic chair protocol may have influenced performance. We did accommodate for this however with our counterbalanced design to negate any potential training effect. It is plausible that CM may be more effective in highly trained resistance trained populations. Differences between fixed range of motion single joint exercises and multi joint exercises employed in the different modalities may have also resulted in the differences between this study and previous trials findings. Although most athletes will likely utilise free weight exercises in their training, the use of an isokinetic chair should be considered a strength for its ability to deliver a consistent resistance and standardisation across subjects.

Citrulline malate supplementation also had no effect on the loss of force production and similar results were noted by Martinez-Sanchez et al. [29], who found CM was ineffective at preserving mean average force, peak average force, or the decline in force production following 8 sets of 8 half squats at 80% RM. The addition of pomegranate juice containing ellagitannins however did improve efficacy of the supplement [29]. Similarly, a recent trial of a pre-workout supplement which compared 3 supplements; one containing BCAA’s, creatine, caffeine and a 6 g dose of CM; one containing the same (but without caffeine); and a placebo, failed to find any significant improvement in loss of force during resistance exercise from either supplement [30]. In contrast, CM was effective in retaining muscle power measured by counter measure jump in runners following a marathon Martinez-Sanchez et al. [29] but not at retaining peak power following maximal cycle sprints in well-trained cyclists, [20] these results suggest the effectiveness of CM to retain power may be dependent exercise modality.

The GVT protocol utilised was relatively novel and although it isn’t generally employed by athletic populations such programmes are common amongst recreational weight trainers likely to utilise and benefit from CM. The protocol effectively reduces the number of repetitions achieved across set and was effective at increasing lactate concentrations in both the placebo and CM group. Citrulline malate however had no effect on lactate buffering when compared to a placebo which might explain the lack of difference in repetition performance between the placebo and CM group. We did not measure citrulline, arginine or NOS, following supplementation. However, previous investigations have demonstrated there is a dose response relationship between ingestion of CM and plasma citrulline, ornithine, arginine and glutamine 1 h after consumption [10, 20, 29]. L-arginine is the key substrate in NO synthesis and it has been suggested that CM may increase NO synthesis indirectly: and subsequently muscle waste-product clearance e.g. lactate [25]. Previous trials have produced mixed results in response to CM on lactate acid. It seems exercise modality may have a bearing on CMs ability to influence blood lactate concentrations. Martinez-Sanchez et al. [29] for example found a reduction in lactate and an increase in lactate dehydrogenase, responsible for converting lactate to pyruvate following marathon running using a Watermelon CM drink containing 3.45 g of citrulline. While, Kiyici et al. [5] reported a reduction in lactate concentrations in handball players immediately following exercise after supplementing with 4 g of CM 4 times per week for 4 weeks. In contrast Cunniffe et al. [20] found no effect of 12 g of CM on blood pH or lactate following 10 sets of 15 s anaerobic sprints on a cycle ergonometer. Likewise, a 6 g per day, 15 supplementation protocol of CM had no effect on muscle pH in patients suffering from fatigue following aerobic finger flexion exercise [2]. Furthermore, in trials similar to this one, Wax et al. [18], Wax et al. [19] and de Silva et al. [25] all found no effect of 6 to 8 g of CM on lactate concentrations following resistance training exercise. While Martinez-Sanchez et al. [29] reported no difference in lactate dehydrogenase concentrations compared to placebo following half-squat exercises. Interestingly however Martinez-Sanchez et al. [29] did report a different in plasma urea concentrations when citrulline was consumed in combination with pomegranate extract high in ellagitannins suggesting CM play some role in ammonia metabolism, proteolysis and recovery.

The eccentric component of exercise is thought to be a prominent factor in causing DOMS [31], despite employing a concentric only protocol, the GVT was effective in increasing muscle soreness following the intervention. The CM did not attenuate the muscle soreness and actually appeared to increase soreness. This lack of effect on muscle soreness is in agreement with other investigations [25], and in contrast to other investigations [17, 29]. Muscle soreness was also significantly reduced following administration with citrulline infused watermelon drinks following both a half marathon [29] and repeated sprints on a cycloergonometer (32). The fact that soreness was reduced following both aerobic and anaerobic exercise trials may make a case for CM being more effective in reducing soreness across modalities. The lack of soreness seen in this study is consistent with the fact we also found no difference in repetition performance, force loss, or lactate between the placebo and CM group. Presumably greater muscle soreness would likely have been observed had more repetitions been achieved from either group, although CM is proposed to offset this soreness by increased ammonium and lactate clearance. We however did not measure ammonium concentrations but did note a failure in CM to resist fatigue.

Chemical analysis of the CM product used in the study suggested that the citrulline:malate composition ratio was lower than that claimed by the manufacturer. The present investigation therefore used a lower dose of citrulline than we had intended administering (around 4.2 g instead of 5.3 g, based on a 2:1 ratio), this is still higher than the dose contained in many pre-exercise supplements and comparable doses administered in other studies [18, 19]. The lack of significant findings may therefore reflect a lower dosing of CM by comparison to other investigations. Interestingly citrulline has been infused into watermelon in other trials [29, 32] and appears to be more readily absorbed from a matrix of watermelon compared to a functional drink. Many manufacturers state the use of 2:1 formulas and it is unclear how much citrulline was administered in other investigations as the CM ratios and chemical analysis is often unreported. Further investigations should therefore explore the composition of the product being tested, as this may influence measurements and outcomes. Citrulline has been purported for its performance enhancing effect, while malate has been cited to improve performance via being an intermediate in the citric acid cycle. It seems more likely that citrulline is the active ingredient as malic acid is utilised to allow the supplement to form a stable salt for storage and neutralise the basicity of the supplement. This stability issue may be overcome by infusion into a watermelon matrix. Performance-enhancing effects have been found from using citrulline in isolation [14] and researchers may wish to concentrate on using pure citrulline, to avoid under dosed products.

Finally, given a number of trials suggesting a positive effect of CM on anaerobic performance (for e.g.’s see [19, 22]) further trials should be carried out utilising both acute and loading doses of CM for both isokinetic and multi joint protocols, using experienced resistance trained populations. There were a number of limitations of the present investigation include no measure of CK or ammonia to accompany the data gathered on lactate and muscle soreness. The subjects who participated in the study were not a homogenous group, and subjects varied in their training experience and included both men and women not controlling for the impact of menstrual cycling; however all participants had at least six months regular resistance training experience and are likely customers for the supplement in question. We also did not perform the experiment fasted, but subjects were instructed to consume the same breakfast on both occasions, however we did note no difference in 24 h dietary intake between laboratory visits and point out that exercisers are unlikely to perform in the fasted state. We also did not control or audit dietary supplements other than asking subjects to not stop or begin supplement regimes throughout the trial the rationale for this is that stopping or starting a new supplement could either positively or negatively affect performance. Furthermore, the isokinetic chair induces an eccentric and concentric force which is not the same, for example, as a standard leg extension exercise performed in a commercial fitness facility; field studies with commonly used equipment and exercises are needed to help answer questions over the efficacy of CM. Additionally particular attention should be paid to the chemical analysis of supplement content carried out in the present paper, which raises concerns over manufacturer’s claims. Athletes and coaches should remain sceptical as to the efficacy of CM.

## Conclusions

Citrulline malate supplementation is purported to improve exercise performance and improve recovery. The findings of this investigation failed to support this claim. Previous studies however have managed to find positive effects of CM supplementation on athletic performance, although this may be limited to high intensity anaerobic resistance training [17, 18]. The ability of CM to increase arginine and potentially influence NOS, ammonia clearance may remain valid, however the recent inconsistent findings in the literature suggest CM ability to influence the aforementioned factors may not result in marked improvements for performance. Although the present investigation adds evidence to the body of literature on CM, further trials are required to test the efficacy of CM supplementation in both acute and loading trials.

## Additional files

Additional file 1: Integration values for all ^1^H chemical environments in citrulline malate, relative to the signal at H9. (PDF 133 kb)

Additional file 2: Integration ratios between all nuclei in citrulline (H5, H2, H4, H3) and those in malate (H8, H9), including mean and standard deviation for individual experiments: values calculated from data in Additional file 1. (PDF 120 kb)

Additional file 3: Mean Number of Repetitions Performed Per Set, with and without Citrulline Malate Supplementation. (PDF 11 kb)

Additional file 4: Quadriceps Muscle Soreness Over Time following German Volume Training with, and without Citrulline Malate. (PDF 9 kb)

Additional file 5: Mean site specific muscle soreness score following German Volume Training. (PDF 7 kb)
