# Supplementary material for: Citrulline malate supplementation does not improve German Volume Training performance or reduce muscle soreness in moderately trained males and females
Source: J Int Soc Sports Nutr. 2018 Aug 10;15:42. doi: 10.1186/s12970-018-0245-8 (PMC6086018; doi:10.1186/s12970-018-0245-8)
Supplement: Supplementary file 1 — Integration values for all 1H chemical environments in citrulline malate, relative to the signal at H9. (PDF 133 kb) [file 12970_2018_245_MOESM1_ESM.pdf]

Additional file 1. Integration values for all ^{1}H chemical environments in citrulline malate, relative to the signal at H9.

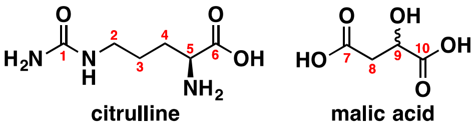

		Citrulline				Malic Acid	
**Sample**	**n**	**H5 (3.65, 1H)**	**H2 (2.96, 2H)**	**H4 (1.65, 2H)**	**H3 (1.43, 2H)**	**H9 (4.37, 1H)**	**H8 (2.70, 2H)**
**A**	**1**	1.6062	3.2810	3.2942	3.3043	1.000	2.0581
	**2**	1.4777	3.0538	3.0847	3.0771	1.000	2.0668
	**3**	1.7590	3.5838	3.5938	3.6102	1.000	2.1116
**B**	**1**	2.0075	3.9889	3.9974	3.9287	1.000	1.9094
	**2**	1.8801	3.7434	3.8589	3.8458	1.000	2.0575
	**3**	1.8700	3.7396	3.7775	3.8043	1.000	2.0650
**C**	**1**	1.4449	2.9574	2.9803	2.9340	1.000	1.9909
	**2**	1.5433	3.0975	3.1774	3.1635	1.000	2.0994
	**3**	1.5452	3.1401	3.2020	3.1722	1.000	2.0940
**D**	**1**	1.6930	3.4156	3.5606	3.4290	1.000	1.9620
	**2**	1.4904	2.9904	3.0342	3.0498	1.000	2.0823
	**3**	1.4699	2.9699	3.0594	3.0269	1.000	2.0451
**E**	**1**	1.1224	2.2566	2.2668	2.2460	1.000	1.9967
	**2**	1.1067	2.2098	2.2494	2.2632	1.000	2.0967
	**3**	1.1107	2.2159	2.2909	2.2559	1.000	2.0640
