# Supplementary material for: Citrulline malate supplementation does not improve German Volume Training performance or reduce muscle soreness in moderately trained males and females
Source: J Int Soc Sports Nutr. 2018 Aug 10;15:42. doi: 10.1186/s12970-018-0245-8 (PMC6086018; doi:10.1186/s12970-018-0245-8)
Supplement: Supplementary file 2 — Integration ratios between all nuclei in citrulline (H5, H2, H4, H3) and those in malate (H8, H9), including mean and standard deviation for individual experiments: values calculated from data in Additional file 1. (PDF 120 kb) [file 12970_2018_245_MOESM2_ESM.pdf]

Additional File 2. Integration ratios between all nuclei in citrulline (H5, H2, H4, H3) and those in malate (H8, H9), including mean and standard deviation for individual experiments: values calculated from data in additional file 1.

		individual nuclear ratios									
**S**	**n**	**H5/H9**	**(H2/2)/ H9**	**(H4/2)/ H9**	**(H3/2)/ H9**	**H5/H8**	**H2/H8**	**H4/H8**	**H3/H8**	**mean**	**st. dev.**
**A**	**1**	1.606	1.641	1.647	1.652	1.561	1.594	1.601	1.606	1.613	0.0311
	**2**	1.478	1.527	1.542	1.539	1.430	1.478	1.493	1.489	1.497	0.0378
	**3**	1.759	1.792	1.797	1.805	1.666	1.697	1.702	1.710	1.741	0.0537
**B**	**1**	2.008	1.994	1.999	1.964	2.103	2.089	2.094	2.058	2.038	0.0535
	**2**	1.880	1.872	1.929	1.923	1.828	1.819	1.876	1.869	1.874	0.0390
	**3**	1.870	1.870	1.889	1.902	1.811	1.811	1.829	1.842	1.853	0.0348
**C**	**1**	1.445	1.479	1.490	1.467	1.452	1.485	1.497	1.474	1.474	0.0183
	**2**	1.543	1.549	1.589	1.582	1.470	1.475	1.513	1.507	1.529	0.0447
	**3**	1.545	1.570	1.601	1.586	1.476	1.500	1.529	1.515	1.540	0.0435
**D**	**1**	1.693	1.708	1.780	1.715	1.726	1.741	1.815	1.748	1.741	0.0403
	**2**	1.490	1.495	1.517	1.525	1.431	1.436	1.457	1.465	1.477	0.0353
	**3**	1.470	1.485	1.530	1.513	1.437	1.452	1.496	1.480	1.483	0.0304
**E**	**1**	1.122	1.128	1.133	1.123	1.124	1.130	1.135	1.125	1.128	0.00488
	**2**	1.107	1.105	1.125	1.132	1.056	1.054	1.073	1.079	1.091	0.0300
	**3**	1.111	1.108	1.145	1.128	1.076	1.074	1.110	1.093	1.106	0.0244
