# Supplementary material for: Citrulline malate supplementation does not improve German Volume Training performance or reduce muscle soreness in moderately trained males and females
Source: J Int Soc Sports Nutr. 2018 Aug 10;15:42. doi: 10.1186/s12970-018-0245-8 (PMC6086018; doi:10.1186/s12970-018-0245-8)
Supplement: Supplementary file 3 — Mean Number of Repetitions Performed Per Set, with and without Citrulline Malate Supplementation. (PDF 11 kb) [file 12970_2018_245_MOESM3_ESM.pdf]

**Additional File 3. Mean Number of Repetitions Performed Per Set, with and without Citrulline Malate Supplementation**

| | Set 1 | Set 2 | Set 3 | Set 4 | Set 5 | Set 6 | Set 7 | Set 8 | Set 9 | Set 10 |
|---|---|---|---|---|---|---|---|---|---|---|
| **Citrulline Malate** | 10.0 ± 0.0 | 10.0 ± 0.0 | 9.7 ± 0.8 | 9.8 ± 0.5 | 9.3 ± 2.0 | 9.4 ± 2.0 | 8.9 ± 2.2 | 8.2 ± 2.2 | 8.2 ± 2.4 | 7.4 ± 2.6 |
| **Placebo** | 10.0 ± 0.0 | 10.0 ± 0.0 | 10.0 ± 0.0 | 9.9 ± 0.3 | 9.8 ± 0.8 | 9.9 ± 1.4 | 8.9 ± 1.5 | 8.8 ± 1.6 | 8.8 ± 1.6 | 8.4 ± 1.8 |
| **Mean reps** | 10.0 ± 0.0 | 10.0 ± 0.0 | 9.7 ± 0.6[ab] | 9.9 ± 0.4 | 9.4 ± 1.5[cde] | 9.4 ± 1.7[fg] | 8.8 ± 1.9 | 8.1 ± 2.0[acf] | 8.0 ± 2.1[d] | 7.7 ± 2.3[beg] |
| **P Value Sets** | | | | | | 0.01 | | | | |
| **P Value Treatment** | | | | | | 0.33 | | | | |
| **P Value Treatment × Sets** | | | | | | 0.34 | | | | |

**Additional File 3. Mean Number of Repetitions Performed Per Set, with and without Citrulline Malate Supplementation.** ± indicates standard deviation, Means were compared using an repeated measures ANOVA. Post Hoc comparison was performed using a pairwise comparison and Bonferroni adjustment. Sets with the same letter are significantly different from one another $P < 0.05$.
