# Supplementary material for: Citrulline malate supplementation does not improve German Volume Training performance or reduce muscle soreness in moderately trained males and females
Source: J Int Soc Sports Nutr. 2018 Aug 10;15:42. doi: 10.1186/s12970-018-0245-8 (PMC6086018; doi:10.1186/s12970-018-0245-8)
Supplement: Supplementary file 4 — Quadriceps Muscle Soreness Over Time following German Volume Training with, and without Citrulline Malate. (PDF 9 kb) [file 12970_2018_245_MOESM4_ESM.pdf]

Additional File 4. **Quadriceps Muscle Soreness Over Time following German Volume Training with, and without Citrulline Malate.**

	Pre	Post	24 h	48 h	72 h
**Citrulline Malate**	14.3 ± 13.7	73.1 ± 31.4	75.8 ± 33.4	65.1 ± 31.1	30.3 ± 19.8
**Placebo**	14.2 ± 12.5	59.7 ± 31.6	56.3 ± 34.0	26.8 ± 16.8	11.3 ± 6.4
**Mean Soreness**	14.2 ± 12.9[a]	66.4 ± 31.7[b]	66.0 ± 34.5[bc]	46.0 ± 31.4[c]	20.9 ± 17.49[a]
**P Value Time**			< 0.01		
**P Value Treatment**			< 0.01		
**P Value Time ×Treatment**			0.01		

Additional File 4. Quadriceps Muscle Soreness Over Time following German Volume Training with, and without Citrulline Malate. ±, indicate standard deviation, means were compared using a repeated measures ANOVA. Post Hoc comparison was performed using a pairwise comparison and Bonferroni adjustment. There was a significant difference between treatment, time and treatment × time $P < 0.01$. Different letters indicate a significant difference between time points. Error bars indicate standard error of the mean, h, hours.
