# Supplementary material for: Citrulline malate supplementation does not improve German Volume Training performance or reduce muscle soreness in moderately trained males and females
Source: J Int Soc Sports Nutr. 2018 Aug 10;15:42. doi: 10.1186/s12970-018-0245-8 (PMC6086018; doi:10.1186/s12970-018-0245-8)
Supplement: Supplementary file 5 — Mean site specific muscle soreness score following German Volume Training. (PDF 7 kb) [file 12970_2018_245_MOESM5_ESM.pdf]

Additional File 5. Mean site specific muscle soreness score following German Volume Training

	Pre	Post	24 h	48 h	72 h
Vastus Medialis	4.1 ± 4.4	25.1 ± 13.0	23.0 ± 15.5	19.0 ± 12.0	9.4 ± 9.4
Vastus Lateralis	4.0 ± 3.6	21.3 ± 12.3	21.3 ± 13.7	18.4 ± 10.2	7.7 ± 7.8
Tensor Fasciae	2.9 ± 2.9	12.1 ± 8.0	16.5 ± 11.6	15.3 ± 11.7	8.2 ± 6.3
Rectus Femoris	3.3 ± 3.5	14.5 ± 9.4	15.3 ± 9.0	12.5 ± 9.8	5.3 ± 4.1

± indicates standard deviation, h hours
